# White matter hyperintensities in dementia with Lewy bodies are associated with poorer cognitive function and higher dementia stages

**DOI:** 10.3389/fnagi.2022.935652

**Published:** 2022-08-26

**Authors:** Tai-Yi Chen, Po-Chi Chan, Ching-Fang Tsai, Cheng-Yu Wei, Pai-Yi Chiu

**Affiliations:** ^1^Department of Radiology, Show Chwan Memorial Hospital, Changhua, Taiwan; ^2^Department of Neurology, Show Chwan Memorial Hospital, Changhua, Taiwan; ^3^Tainan Sin-Lau Hospital, The Presbyterian Church in Taiwan, Tainan, Taiwan; ^4^Department of Neurology, Chang Bing Show Chwan Memorial Hospital, Changhua, Taiwan; ^5^Department of Applied Mathematics, Tunghai University, Taichung, Taiwan

**Keywords:** white matter hyperintensities, dementia with Lewy bodies, Fazekas, cognitive, sums of boxes of the clinical dementia rating, cognitive abilities screening instruments

## Abstract

**Purpose:**

White matter hyperintensities (WMHs) are frequently found in elderly individuals with or without dementia. However, the association between WMHs and clinical presentations of dementia with Lewy bodies (DLB) has rarely been studied.

**Methods:**

We conducted a retrospective analysis of patients with DLB registered in a dementia database. WMHs were rated visually using the Fazekas scale, and its associated factors including dementia severity, cognitive functions, neuropsychiatric symptoms, and core clinical features were compared among different Fazekas scores. Domains in the Clinical Dementia Rating (CDR), Cognitive abilities Screening Instruments (CASI), and Neuropsychiatric Inventory (NPI) were compared among different Fazekas groups after adjusting for age, sex, education, and disease duration.

**Results:**

Among the 449 patients, 76, 207, 110, and 56 had Fazekas score of 0, 1, 2, and 3, respectively. There was a positive association between dementia severity and WMHs severity, and the mean sums of boxes of the Clinical Dementia Rating (CDR-SB) were 5.9, 7.8, 9.5, and 11.2 (*f* = 16.84, *p* < 0.001) for the Fazekas scale scores 0, 1, 2, and 3, respectively. There was a negative association between cognitive performance and WMHs severity, and the mean CASI were 57.7, 45.4, 4.06, and 33.4 (*f* = 14.22, *p* < 0.001) for the Fazekas scale scores 0, 1, 2, and 3, respectively. However, WMHs were not associated with the core clinical features of DLB. After adjustment, all cognitive domains in CDR increased as the Fazekas score increased. In addition, performance on all cognitive domains in CASI decreased as the Fazekas score increased (all *p* < 0.001). Among neuropsychiatric symptoms, delusions, euphoria, apathy, aberrant motor behavior, and sleep disorders were significantly worse in the higher Fazekas groups compared to those in the group with Fazekas score of 0 after adjustment.

**Conclusion:**

WMHs in DLB might contribute to deterioration of cognitive function, neuropsychiatric symptoms, and dementia stages. However, core clinical features were not significantly influenced by WMHs in DLB.

## Introduction

White matter hyperintensities (WMHs) resulting from chronic ischemia associated with cerebral small vessel disease are frequently detected on magnetic resonance imaging (MRI) in elderly individuals (Prins et al., 2004). WMHs predict an increased risk of stroke, dementia, and death; therefore, detailed screening for risk factors of stroke and dementia should be performed if WMHs were identified (Debette and Markus, 2010). The histopathology of WMHs is various. The tissue damages range from slight disentanglement of the matrix to myelin and axonal loss (Prins and Scheltens, 2015; Wardlaw et al., 2015). In a previous study, WMHs were more frequent in patients with dementia than in patients with normal cognition (Sarro et al., 2016) and were strongly associated with cognitive dysfunction, especially in processing speed or executive functions (de Groot et al., 2000; Prins et al., 2004; Burton et al., 2006; Defrancesco et al., 2013). In patients with mild cognitive impairment (MCI), low scores in orientation and verbal delayed recall were predictors of progression from MCI to Alzheimer’s disease (AD) (Defrancesco et al., 2013). WMHs can be used to predict an increased risk of dementia (Wolf et al., 2000; van Straaten et al., 2008; Brickman et al., 2012; Kamagata et al., 2013) and a high WMHs burden may result in imminent progression from MCI to dementia (de Groot et al., 2000).

Previous studies have focused on the association between WMHs and cerebrovascular disease or dementia due to AD (Wolf et al., 2000; Defrancesco et al., 2013; Taylor et al., 2017). However, the association or influence of WMHs burden on the clinical manifestation or progression of dementia with Lewy bodies (DLB) has less been studied. DLB is the second most common degenerative dementia, accounting for 0–26.3% of hospital- or population-based dementia cases (Zaccai et al., 2005). In a previous study, patients with DLB presented with fewer WMHs than those with vascular dementia (Barber et al., 1999; Ihara et al., 2010), while those with DLB and AD showed more severe WMHs than patients with Parkinson’s disease (PD) with dementia (Joki et al., 2018). According to a previous study, WMHs in AD and DLB may be determined by similar processes and periventricular hyperintensities, but not deep WMHs, independently correlate with advancing age and increasing ventricular dilatation (Barber et al., 2000). However, studies investigating the contribution of WMHs or its associated factors in DLB have shown greater occipital WMHs in patients with DLB than in those with AD (Watson et al., 2012; Nedelska et al., 2015; Sarro et al., 2016). In addition, another study has reported an inverse relationship between small vessel disease scores and the severity of Lewy pathology in patients with Lewy body disease (LBD) (Ghebremedhin et al., 2010). There was an increased severity of WMHs on MRI, but not neuropathology, in DLB and Parkinson’s disease (PD) with dementia compared to PD without dementia and age-matched controls (Hijazi et al., 2022).

Some studies have reported an association between cognitive function and WMHs in patients with PD or DLB. One study showed a significant correlation between WMHs and Mini-Mental State Examination and verbal fluency scores in the AD group, but not in the DLB group (Oppedal et al., 2012). Another found a significant association between increased total WMHs volume and worse performance in executive function, memory, and language (Vesely and Rektor, 2016).

The Fazekas scale is a simple visual rating tool for clinical assessment of WMHs in normal aging and dementia (Fazekas et al., 1987). To investigate the factors associated with WMHs in patients with DLB, the Fazekas scale was used in a relatively large sample size of patients with DLB. The important clinical features, cognitive performance, motor dysfunction including gait disturbance, neuropsychiatric symptoms, medication, and vascular risk factors (VRFs) were analyzed and compared between the high and low Fazekas groups. Based on the current evidence, we hypothesized that higher WMHs should have higher dementia severity, WMHs in DLB might contribute to more cognitive and motor dysfunction, and worse neuropsychiatric symptoms.

## Materials and methods

### Database

This retrospective study included patients aged more than 59 years old with probable DLB registered in the dementia database of the Show Chwan Health Care System. Registration is currently ongoing in three hospitals in Taiwan. The Committee for Medical Research Ethics of Show Chwan Memorial Hospital reviewed the project, and the Data Inspectorate approved it (SCMH_IRB No: IRB1081006). The following information was extracted from this database and used in this study:

(1) Diagnosis of dementia (major neurocognitive disorder) according to the criteria for primary degenerative dementia in the fifth edition of the Diagnostic and Statistical Manual of Mental Disorders. Diagnosis of DLB according to the revised consensus criteria for probable or possible DLB developed by the fourth report of the DLB consortium (McKeith et al., 2017).

(2) Age, sex, education, dementia severity according to the Clinical Dementia Rating Scale (CDR) and CDR-SB, such as memory, orientation, judgment, community affairs, home hobbies, and personal care (Morris, 1997), and motor severity assessed using the motor score (UPDRS-m) and gait sub-score (UPDRS-gait) of the Unified Parkinson’s Disease Rating Scale (UPDRS) (Ballard et al., 1997; Movement Disorder Society Task Force on Rating Scales for Parkinson’s, 2003) at the time of entry.

(3) Clinical features of DLB, including fluctuation in cognition, parkinsonism, visual hallucinations (VH), rapid eye movement sleep behavior disorder (RBD), and abnormal dopamine transporter imaging in partial participants (McKeith et al., 2017). To acquire a detailed clinical history, all participants were asked to complete a registration form, including a detailed history registration questionnaire, named the History-based Artificial Intelligent Clinical Dementia Diagnostic System (HAICDDS) (Lin et al., 2018; Chiu et al., 2019; Wang et al., 2020). Prior to starting the study, we trained 12 neuropsychologists in three centers, and reliability tests for HAICDDS and neuropsychological tests were performed after training. The HAICDDS is a structured questionnaire used for the diagnosis of core clinical features of DLB. It includes 6 domains on fluctuation, 7 domains on parkinsonism, 2 domains on VH, and 4 domains on RBD. Fluctuations were diagnosed when a mayo fluctuation composite score of > 2 and a clinical history of fluctuation in cognition were present; VH were diagnosed when a clinical history of recurrent well-formed, complex, and detailed VH was present. Parkinsonism was diagnosed when at least two of the following were present: bradykinesia, rigidity, tremor, and postural instability; RBD was diagnosed when the minimum criteria for RBD according to the International Classification of Sleep Disorders were met (American Academy of Sleep Medicine, 2005).

(4) Cognitive performance assessed using the Chinese version of the Cognitive Abilities Screening Instrument (CASI) with the following domains: remote memory, recent memory, attention, mental manipulation, orientation, abstract thinking, language, drawing, and verbal fluency (Teng et al., 1994). Montreal Cognitive Assessment (MoCA) includes the following domains: visuospatial/executive, naming, attention, language, abstraction, memory, and orientation (Nasreddine et al., 2005; Kasten et al., 2010). Activities of daily living (ADL) assessed using the ADL scale embedded in the HAICDDS questionnaire (HAIADL) (Hung et al., 2021).

(5) Composite scores of the neuropsychiatric symptoms in the 12-item version of the NPI, such as delusions, hallucinations, agitation, depression, anxiety, euphoria, apathy, disinhibition, irritation, aberrant motor behavior, night behavior, and eating/appetite behavior. They were monitored according to observations in the past month (Cummings et al., 1994).

(6) Information on clinically relevant vascular risk comorbidities including hypertension, diabetes, hyperlipidemia, coronary artery disease, arrhythmia, congestive heart failure, and cerebrovascular disease (history of stroke or the diagnosis of vascular encephalopathy in brain imaging).

(7). MRI and WMHs ratings: MRI was performed using one of the four MRI scans currently used in the three centers (one 1.5 T Ingenia, Philips Healthcare, Best, the Netherlands, one 1.5 T Avanto, Siemens Medical Solutions, Erlangen, Germany, one 1.5 T Optima 450W, GE Healthcare, Milwaukee, Wisconsin, United States; and one 1.5 T Signa EXCITE-HD, GE Healthcare, Milwaukee, Wisconsin, United States). They were obtained at first clinical workup. The Fazekas scale (Fazekas et al., 1987) was used to determine the severity of WMHs. A total Fazekas WMH score, usually ranging from 0 to 6, was obtained by summing the periventricular (PVWM) and deep white matter (DWM) scores. In this study, to simplify the categories, we combined the periventricular and DWM scores and reduced the categories to 0–3. If PVWM and DWM scores were different, higher ones were selected for categorizing. Protocols include axial DWI (*b*-value 0, 1,000 s/mm^2^) and ADC map, axial FLAIR (TE: 110 ms, TR: 9,000 ms, Flip angle (FA): 90, inversion time: 2,400 ms, FOV: 14 × 23 cm, slice thickness: 5 mm), axial T1WI (TE: 13 ms, TR: 450 ms, FA: 90, FOV: 14 × 23 cm, slice thickness: 5 mm), axial T2WI (TE: 85 ms, TR: 5,000 ms, FA: 90, FOV: 14 × 23 cm, slice thickness: 5 mm), axial SWI (3D-MRA TOF without contrast), sagittal 3D-T1 SPGR (fast spoiled gradient-recalled echo), and coronal T2WI (TE: 85 ms, TR: 3,500 ms, FA: 150, FOV: 20 × 20 cm, SL: 3 mm). The key sequence for evaluating the severity of WMHs is axial FLAIR sequence. Before the starting of the study, 36 patients were tested by one neuroradiologist (TY Chen) and one neurologist (PY Chiu) and the reproducibility was studied using the interrater reliability analysis with a high intra-class correlation coefficient of 0.927.

(8) Striatal background ratio and caudate putamen ratio (CPR) were assessed using Tc-99m Trodat-1 SPECT and analyzed semi-quantitatively (Huang et al., 2012). Dopamine transporter imaging is one of the indicative biomarkers; therefore, some participants received 99m Trodat-1 SPECT or cerebral perfusion SPECT to obtain more accurate diagnosis. The procedure of the dopamine transporter imaging is as the following: A dose of 25 mCi of [99 mTc] TRODAT-1 was injected intravenously into each patient. The binding to dopamine transporter was assessed 4 h after injection by SPECT. A rotating three-headed gamma camera with fan-beam collimator (Multi SPECT 3, Siemens, Germany) and a commercially available computer system were used for data acquisition and processing. Data were collected for 120 projections (360° rotation) in a 128 × 128 matrix. The acquisition time was 40 s per projection. Attenuation correction was performed in the selected transverse slices according to a modified Chang’s method. In-plane resolution of the reconstructed images was 8.5 mm FWHM, and slice thickness was approximately 6 mm.

### Data analysis

The Chinese version of SPSS 22.0 for Windows (IBM, SPSS Inc., Chicago) was used for statistical analyses. Background characteristics, clinical features, VRFs, current medication usage, performance on different cognitive domains, and manifestation of neuropsychiatric symptoms of patients with DLB in different Fazekas groups were compared. Comparison of characteristics included age, education level, global dementia severity according to the CDR and CDR-SB, results of cognitive tests including subscale scores of the CASI and MoCA, and subscale scores of the neuropsychiatric symptoms according to the NPI. One-way ANOVA with either Bonferroni or Dunnett T3 *post hoc* analysis was according to the homogeneity of variance. Sex, clinical features of DLB, VRFs, and current medication usage were analyzed using the chi-square test with Bonferroni correction. The motor scores of the UPDRS and findings of dopamine transporter imaging performed using Tc-99m Trodat-1 SPECT were analyzed among the partial participants (36, 86, 42, and 22 cases with Fazekas 0, 1, 2, and 3, respectively). Domains in the CDR including a supplementary domain of language (HAICDDS-Language, Lin et al., 2018), CASI, and NPI were compared using multivariate logistic regression among different Fazekas group after adjusting for age, sex, education, and disease duration. Odds ratio (OR) of the higher Fazekas groups were compared to that of the group with Fazekas score of 0.

## Results

The registration period for this study was from September 2015 to August 2020. During this period, we registered 9,607 individuals with either normal cognition (*n* = 2,131), MCI (*n* = 2,485), or dementia (*n* = 4,269) in a hospital-based cohort. In the dementia group, 1,841 (47.1%) patients had probable AD, 449 (10.5%) patients had DLB, 1,979 (43.4%) patients had other subtypes of dementia or undetermined dementia. Among patients with DLB, 76, 207, 110, and 56 patients had Fazekas scale scores of 0, 1, 2, and 3, respectively (Table 1). A comparison of ages among the four Fazekas subgroups revealed no significant association, and the mean age was 78.6, 79.5, 80.8, and 80.5 for the Fazekas scale scores of 0, 1, 2, and 3, respectively. The sex disparity, education, and disease duration were not significantly different among the four Fazekas subgroups, either. However, there was a positive association between dementia severity and WMH severity, and the mean

**TABLE 1:** Comparison of demographical characteristics among different Fazekas groups.

	Fazekas 0 (F0) Mean (SD, range)	Fazekas 1 (F1) Mean (SD, range)	Fazekas 2 (F2) Mean (SD, range)	Fazekas 3 (F3) Mean (SD, range)	*F*/X^2^	*p*	*Post hoc*
*N*	76	207	110	56			
Age, year	78.6 (5.1, 66–93)	79.5 (6.7, 60–97)	80.8 (6.8, 61–95)	80.5 (7.3, 60–97)	2.01	NS	
Female, *n*	31 (40.8)	100 (48.3)	52 (47.3)	31 (55.4)	2.81	NS	
Education, year	4.7 (4.0, 0–16)	4.1 (4.3, 0–18)	3.4 (3.9, 0–14)	3.5 (4.2, 0–16)	1.64	NS	
Duration, year	1.9 (1.9, 0.2–10.0)	2.3 (2.5, 0.2–13.0)	2.0 (2.3, 0.2–15.0)	2.8 (3.3, 0.3–16.0)	1.99	NS	
CDR	1.0 (0.7, 0.5–3.0)	1.3 (0.8, 0.5–3.0)	1.6 (0.9, 0.5–3.0)	1.8 (0.7, 0.5–3.0)	13.34	<0.001	F0 < F1 < F2 = F3
CDR-SB	5.9 (4.3, 0.5–17.0)	7.8 (4.8, 0.5–18.0)	9.5 (5.1, 1.0–18.0)	11.2 (4.0, 1.0–18.0)	16.84	<0.001	F0 < F1 < F2 = F3
MoCA	10.9 (6.5, 0–24)	7.5 (6.0, 0–28)	6.3 (5.1, 0–19)	5.2 (4.8, 0–22)	13.20	<0.001	F0 > F1 > F3
CASI	57.7 (21.8, 0–93)	45.4 (23.2, 0–94)	40.6 (22.9, 0–82)	33.4 (21.4, 0–79)	14.22	<0.001	F0 > F1 > F3
HAIADL	11.0 (7.8, 2–31)	14.7 (8.3, 2–31)	17.8 (8.4, 2–31)	20.3 (8.0, 2–31)	17.02	<0.011	F0 < F1 < F2 = F3
NPI	9.7 (9.1, 0–41)	14.1 (15.2, 0–101)	15.2 (13.7, 0–90)	17.4 (14.5, 0–72)	3.84	0.010	F0 < F1 = F2 = F3
UPDRS-m	27.2 (12.6, 2–56)	34.2 (19.6, 5–84)	33.4 (21.1, 0–91)	47.4 (18.6, 9–84)	4.81	0.003	F0 = F1 = F2 < F3
UPDRS-gait	1.4 (1.1, 0–4)	1.6 (1.1, 0–4)	1.8 (1.3, 0–4)	2.1 (1.3, 0–4)	4.50	0.004	F0 = F1 = F2 < F3
PVWM, 0/1/2/3	76/0/0/0	2/205/0/0	0/8/102/0	0/0/2/53	1247.5	<0.001	F0 < F1 < F2 < F3
DWM, 0/1/2/3	76/0/0/0	4/203/0/0	1/16/93/0	0/1/4/50	1146.1	<0.001	F0 < F1 < F2 < F3
Cortical MB, *n* (%)	5 (6.6)	14 (6.8)	19 (17.1)	18 (32.7)	31.46	<0.001	F0 = F1 < F2 < F3
Subcortical MB, *n* (%)	9 (11.8)	19 (9.2)	21 (18.9)	17 (30.9)	18.63	<0.001	F0 = F1 < F2 < F3
SBR	1.2 (0.4, 0.2–1.8)	1.2 (0.5, 0.0–2.3)	1.1 (0.4, 0.2–2.2)	1.0 (0.5, 0.0–2.0)	1.11	NS	
CPR	1.7 (0.2, 1.3–2.3)	1.7 (0.3, 1.1–2.4)	1.50 (0.3, 1.0–2.2)	1.5 (0.3, 1.1–2.1)	4.44	0.005	F0 = F1 < F2 = F3

N, Number of cases; Fazekas, Fazekas scale for white matter hyperintensities; NS, Non-significance. Duration, Disease duration; CDR, Clinical Dementia Rating scale; CDR-SB, sum of boxes of the CDR; MoCA, Montreal Cognitive Assessment; CASI, Cognitive Abilities Screening Instrument; HAIADL, History-based Artificial Intelligence Activities of Daily Living scale; NPI, total score of 12-domain Neuropsychiatric Inventory; UPDRS-m, motor score of the Unified Parkinson’s Disease Rating Scale; UPDRS-gait, gait sub-score of the UPDRS-m; PVWM, periventricular white matter score in Fazekas; DWM, deep white matter score in Fazekas; MB, microbleeds; SBR, Striatal background ratio in Tc-99m Trodat-1 SPECT among 36, 86, 42, and 22 cases with Fazekas 0, 1, 2, and 3, respectively; CPR, caudate putamen ratio in Tc-99m Trodat-1 SPECT among 36, 86, 42, and 22 cases with Fazekas 0, 1, 2, and 3, respectively.

CDR-SB were 5.9, 7.8, 9.5, and 11.2 (*f* = 16.84, *p* < 0.001) for the Fazekas scale scores 0, 1, 2, and 3, respectively. There was a negative association between cognitive performance and WMH severity, and the mean CASI were 57.7, 45.4, 4.06, and 33.4 (*f* = 14.22, *p* < 0.001) for the Fazekas scale scores 0, 1, 2, and 3, respectively. In addition, there was a negative association between ADL function and WMH severity, and the mean HAIADL were 11.0, 14.7, 17.8, and 2.3 (*f* = 17.02, *p* < 0.001) for the Fazekas scale scores 0, 1, 2, and 3, respectively. Poorer motor and gait functions according to UPDRS-m (*f* = 4.81, *p* = 0.003) and UPDRS-gait (*f* = 4.50, *p* = 0.004) were found only in the Fazekas 3. The CPR according to Tc-99m Trodat-1 SPECT was significantly higher in the lower Fazekas subgroups (Fazekas 0 and 1) compared to those in the higher Fazekas groups (Fazekas 2 and 3).

Comparisons of core clinical features for the diagnosis of DLB, VRFs, and current medications among different Fazekas groups are summarized in Table 2. Among all participants, frequencies of core clinical features including fluctuation in cognition, VH, parkinsonism, and RBD were 67.3, 43.2, 87.5, and 45.9%, respectively. Frequency of abnormal dopamine transporter imaging was 82.3% (153 with abnormal finding within 186 participants that performed dopamine transporter imaging). Comparison of these important diagnostic features for DLB were not significantly different among the Fazekas groups. Comparisons of VRFs and current medications usage among different Fazekas groups demonstrated that no significant difference among groups after Bonferroni correction except for CVD was higher in the Fazekas 3 group compared to those in the Fazekas 0 or 1 groups (*f* = 14.40, *p* = 0.002).

**TABLE 2:** Comparison of clinical features, vascular risk factors, and current medication usage among different Fazekas groups.

	Fazekas 0 (F0) *n* (%)	Fazekas 1 (F1) *n* (%)	Fazekas 2 (F2) *n* (%)	Fazekas 3 (F3) *n* (%)	X*^2^*	*P*	Bonferroni correction
*n*	76	207	110	56			
**Clinical features**							
Fluctuation	50 (65.8)	131 (63.9)	75 (58.2)	45 (80.4)	5.53	NS	
VH	25 (32.9)	88 (42.5)	52 (47.3)	29 (51.8)	5.76	NS	
Parkinsonism	70 (92.1)	186 (89.9)	89 (80.9)	48 (85.7)	7.07	NS	
RBD	37 (48.7)	101 (48.8)	50 (45.5)	18 (32.1)	5.21	NS	
DaTabN	28 (77.8)	69 (80.2)	39 (92.9)	17 (77.3)	4.35	NS	
**Vascular risk factors**							
CVD	4 (5.3)	21 (10.1)	19 (17.3)	14 (25.0)	14.40	0.002	F0 < F3; F1 < F3
Hypertension	27 (35.5)	93 (45.8)	59 (55.1)	27 (50.9)	7.31	NS	
Diabetes	19 (25.0)	53 (26.1)	29 (27.1)	9 (17.0)	2.20	NS	
Hyperlipidemia	6 (7.9)	38 (18.7)	14 (13.1)	8 (15.1)	5.51	NS	
Heart diseases	15 (19.7)	37 (18.2)	18 (16.8)	8 (15.1)	0.56	NS	
**Current medication**							
Anti-dementia drugs	7 (9.2)	30 (14.5)	13 (11.8)	9 (16.1)	1.95	NS	
Anti-Parkinson drugs	19 (25.0)	39 (18.8)	13 (11.8)	11 (19.6)	5.49	NS	
Antiplatelets	13 (17.1)	40 (19.3)	18 (16.4)	11 (19.6)	3.84	NS	
Antipsychotics	6 (7.9)	27 (13.0)	11 (10.0)	10 (17.9)	3.66	NS	

NS, Non-significance; VH, visual hallucinations; RBD, REM sleep behavior disorder; DaTabN, abnormal dopamine transporter imaging in Tc-99m Trodat-1 SPECT among 36, 86, 42, and 22 cases with Fazekas 0, 1, 2, and 3, respectively; CVD, cerebrovascular disease or transient ischemic attack according to clinical history or MRI imaging; Heart diseases including carotid artery disease, congestive heart failure, and arrhythmia.

**FIGURE 1:**
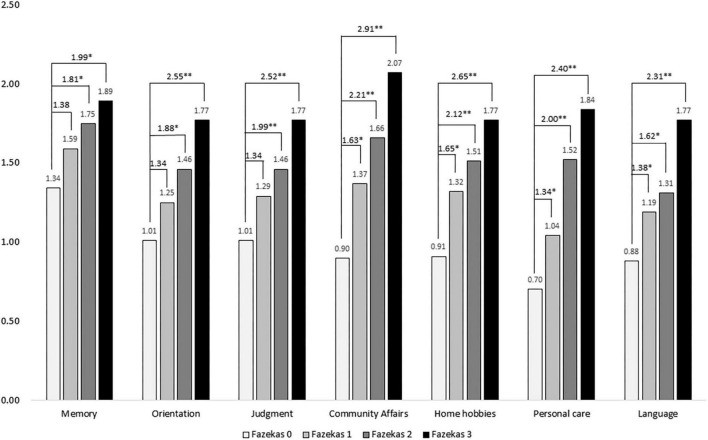
Comparison of scores of boxes of the Clinical Dementia Rating (CDR-SB) among different Fazekas groups adjusted for age, sex, education, and disease duration. ORs were compared to Fazekas score of 0. *p < 0.05; ^**^p < 0.001.

Figure 1 demonstrated a comparison of domains in CDR among different Fazekas groups and showed positive correlations of domains including memory, orientation, judgment, community affairs, home hobbies, personal care, and language with all *p* < 0.001. After adjustment for age, sex, education, and disease duration, all cognitive domains increased as the Fazekas score increased. After adjustment, compared to Fazekas 0 in memory, the ORs were 1.38, 1.81, and 1.99 in Fazekas 1, 2, and 3, respectively. In orientation, the ORs were 1.34, 1.88, and 2.55 in Fazekas 1, 2, and 3, respectively. In judgment, the ORs were 1.34, 1.99, and 2.52 in Fazekas 1, 2, and 3, respectively. In community affairs, the ORs were 1.63, 2.61, and 2.91 in Fazekas 1, 2, and 3, respectively. In home hobbies, the ORs were 1.65, 2.12, and 2.65 in Fazekas 1, 2, and 3, respectively. In personal care, the ORs were 1.34, 2.00, and 2.40 in Fazekas 1, 2, and 3, respectively. In language, the ORs were 1.38, 1.62, and 2.31 in Fazekas 1, 2, and 3, respectively.

**FIGURE 2:**
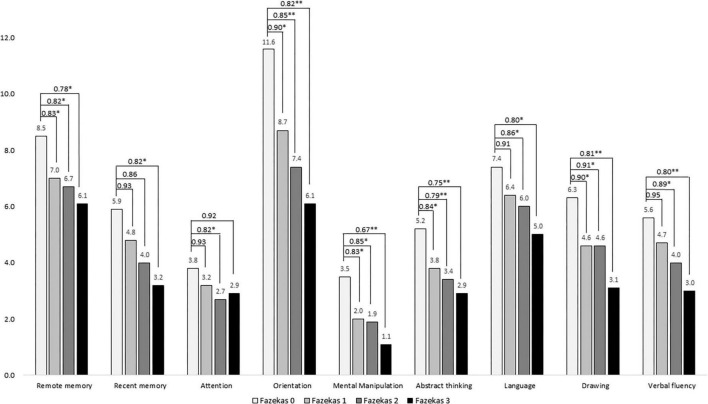
Comparison of cognitive domains of the CASI among different Fazekas groups adjusted for age, sex, education, and disease duration. ORs were compared to Fazekas score of 0. *p < 0.05; ^**^p < 0.001.

Figure 2 demonstrated a comparison of cognitive domains of the CASI among different Fazekas groups and showed positive correlations of domains including remote memory, recent memory, attention, mental manipulation, orientation, abstract thinking, language, drawing, and verbal fluency with all *p* < 0.001. Performance on all cognitive domains decreased as the Fazekas score increased. After adjustment, in remote memory, the ORs were 0.83, 0.82, and 0.78 in Fazekas 1, 2, and 3, respectively. In recent memory, the ORs were 0.93, 0.86, and 0.82 in Fazekas 1, 2, and 3, respectively. In attention, the ORs were 0.93, 0.82, and 0.92 in Fazekas 1, 2, and 3, respectively. In orientation, the ORs were 0.90, 0.85, and 0.82 in Fazekas 1, 2, and 3, respectively. In mental manipulation, the ORs were 0.83, 0.85, and 0.67 in Fazekas 1, 2, and 3, respectively. In abstract thinking, the ORs were 0.84, 0.79, and 0.75 in Fazekas 1, 2, and 3, respectively. In language, the ORs were 0.90, 0.85, and 0.82 in Fazekas 1, 2, and 3, respectively. In drawing, the ORs were 0.90, 0.91, and 0.81 in Fazekas 1, 2, and 3, respectively. In verbal fluency, the ORs were 0.95, 0.89, and 0.80 in Fazekas 1, 2, and 3, respectively.

**FIGURE 3:**
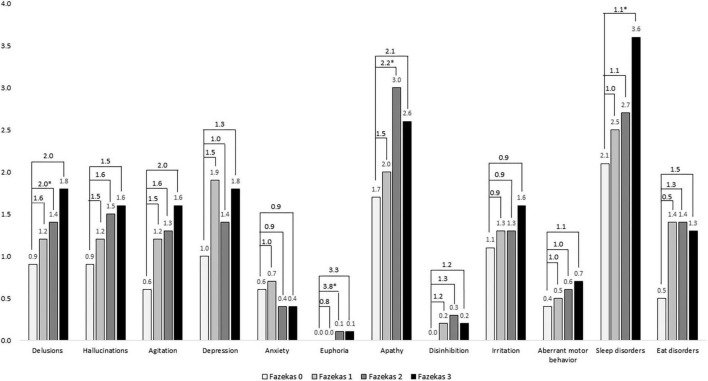
Comparison of composite scores of neuropsychiatric symptoms in the NPI among different Fazekas groups adjusted for age, sex, education, and disease duration. ORs were compared to Fazekas score of 0. *p < 0.05.

Figure 3 shows a comparison of composite scores of neuropsychiatric symptoms in the NPI among different Fazekas groups. Among them, delusions, euphoria, apathy, aberrant motor behavior, and sleep disorders were significantly worse in the higher Fazekas (2 or 3) groups compared to those in the group with Fazekas score of 0 after adjustment. In delusions, the ORs were 1.6, 2.0, and 2.0 in Fazekas 1, 2, and 3, respectively. In euphoria, the ORs were 0.8, 3.8, and 3.3 in Fazekas 1, 2, and 3, respectively. In apathy, the ORs were 1.5, 2.2, and 2.1 in Fazekas 1, 2, and 3, respectively. In sleep disorders, the ORs were 1.0, 1.1, and 1.1 in Fazekas 1, 2, and 3, respectively.

## Discussion

Our study reports some important findings. First, the age, sex disparity, education, and disease duration in this study were not significantly different among the four Fazekas subgroups; however, WMHs were positively correlated with dementia severity and negatively correlated with cognitive performance and ADL function which are consistent with the results of most previous studies on WMHs and dementia (Prins et al., 2004; Sarro et al., 2016). Dopamine transporter imaging is one of the indicative biomarkers for the diagnosis of DLB. In this study, there is no difference in the SBR (striatal background ratio) among different Fazekas groups; however, the CPR is significantly lower in the higher Fazekas groups (Fazekas 2 and 3) indicating a worse cognitive function instead of motor function in the higher Fazekas groups compared to the lower Fazekas groups (Fazekas 0 and 1). This is also compatible with clinical findings of higher dementia severity and lower cognitive performance in the higher Fazekas groups.

Second, we found that there was no significant association between WMHs and core clinical features for the diagnosis of DLB including fluctuation, VH, parkinsonism, and RBD. However, significantly poorer motor and gait functions according to motor and gait sub-scores found in the Fazekas 3 indicated a potential contribution of severe WMHs to motor dysfunction in DLB. In addition, most of the VRFs and current medication usage were not different among Fazekas groups, either. These results suggest that cerebral or systemic

vascular factors contribute less to the emergence of the clinical presentation in DLB. However, in this study, we found a positive association between WMHs and CVD (5.3, 10.1, 17.3, and 25.3% for the Fazekas scale scores of 0, 1, 2, 3, respectively) after Bonferroni correction. This finding is also consistent with the evidence of an inverse relationship between small vessel disease and the severity of cerebral vascular pathology in patients with vascular dementia (Barber et al., 1999; Ihara et al., 2010).

Third, although cognitive screening tests (such as the CASI and MoCA) were used to study the association between cognitive function and WMHs. After adjustment for age, sex, education, and disease duration, patients with DLB and higher Fazekas scale scores had poorer cognitive functions in all domains of CASI including remote memory, recent memory, attention, mental manipulation, orientation, abstract thinking, language, drawing, and verbal fluency than those with lower Fazekas scale scores. Based on the consensus criteria, cognitive deficits in the domains of executive function, visuospatial function, and attention are considered essential for the diagnosis of DLB (McKeith et al., 2017). Therefore, in this study, most cognitive domains in which WMHs contributed to the clinical presentation of DLB were not only the essential cognitive features (visuospatial function, attention, mental manipulation, abstract thinking, and verbal fluency) for the diagnosis of DLB but were also some other cognitive features (orientation, language, remote memory, and recent memory). The poor performance of the higher Fazekas groups could be attributed to the additional effects of WMHs on cognitive dysfunction in the patients with DLB.

Finally, when studying the association between WMHs and neuropsychiatric features in DLB, we found that among neuropsychiatric symptoms, delusions, euphoria, apathy, aberrant motor behavior, and sleep disorders were significantly worse in the higher Fazekas groups compared to those in the group with the Fazekas score of 0 after adjustment. This finding suggests that WMHs might have partial contribution to the neuropsychiatric manifestation of DLB. For example, according to the previous studies and consensus criteria, sleep disorders are striking features of DLB (Yamane et al., 2011; Chiu et al., 2017; McKeith et al., 2017). These findings also support the previous findings that WMHs cannot be considered as mere incidental findings, at least in patients who show severe lesions (Pantoni et al., 2007).

This study has some limitations. First, it was conducted in three hospitals in Taiwan. Therefore, the study findings may not be generalizable to all patients with DLB. Second, the comparison of the associated factors between the high and low Fazekas groups was cross-sectional. Therefore, causal relationships between the factors and dementia could not be ascertained. Third, the current study did not compare DLB with non-synucleinopathy disease; therefore, both contribution of synucleinopathy to deterioration of cognition and interaction of synucleinopathy with WMH were not able to be well demonstrated. In addition, this study did not include the CHIPS score for analysis. According to the previous studies, cholinergic function decline was not only found in the patients with AD, but was also noticed in the patients with Lewy body dementia including DLB and PDD (Francis and Perry, 2007). Fourth, the identification of RBD or DLB type VH is relatively strict in our cohort. Therefore, the prevalence of DLB core clinical features in our cohort was relatively lower. We considered a relatively strict evaluation of core clinical features and neuropsychiatric symptoms to guarantee a better discrimination of DLB from AD. Lastly, only 41.4% study participants had undergone dopamine transporter uptake imaging; this may have resulted in a lower diagnostic rate of probable DLB in this study.

## Conclusion

In conclusion, we evaluated WMHs using brain MRI in a relatively large sample of patients with DLB and performed multi-dimensional analysis of the associated factors of WMHs in DLB according to different Fazekas scale scores. Compared to the lower Fazekas group, the higher Fazekas groups had more severe stages of dementia, poorer cognitive function and ADL, and more severe sleep disorders. There was no significant association between WMHs and core clinical features for the diagnosis of DLB including fluctuation, VH, parkinsonism, and RBD. This cohort will be prospectively followed up for the influence of WMHs on the progression of clinical manifestations of DLB.

## Data availability statement

The original contributions presented in this study are included in the article/supplementary material, further inquiries can be directed to the corresponding author/s.

## Ethics statement

The studies involving human participants were reviewed and approved by the Show Chwan Memorial Hospital. Written informed consent for participation was not required for this study in accordance with the national legislation and the institutional requirements.

## Author contributions

P-YC: conception and study design. C-FT and P-YC: statistical analysis. T-YC: drafting the manuscript work. T-YC, P-CC, and P-YC: revising the manuscript. All authors: data collection and acquisition, interpretation of results, and approval of final version.
